# Exploring the impact of different definitions of level 1 and level 2 sensor‐detected hypoglycaemia upon frequency of hypoglycaemia and time below range: The Hypo‐METRICS study

**DOI:** 10.1111/dom.70238

**Published:** 2025-10-27

**Authors:** Vaios Koutroukas, Jonah Thomas, Gilberte Martine‐Edith, Patrick Divilly, Claire L. Meek, Frans Pouwer, Stephanie A. Amiel, Pratik Choudhary, the Hypo‐METRICS Consortium, Andrew Pernet, Amy Aldridge, Marcia Henderson‐Wilson, Pernille Banck‐Petersen, Rikke Carstensen, Charlotte Hansen, Sharon Caunt, Sue Hudson, Chloe Husband, Fahad Arshad, Anne Farret, Anne‐Marie Marteil‐Oudrer, Manal Shbat, Lisa Crespy, Jenny Constanza Thibout, Anja Rasing, Jesper Walburgh Schimidt, Daniel Loewenstein, Renate Huijsmans, Pieter Drijver, Daniel Chapman, Jane Kennet, Anna Barnett, Jackie Lindsay

**Affiliations:** ^1^ Diabetes Research Centre College of Life Sciences, University of Leicester Leicester UK; ^2^ Leicester Diabetes Centre Leicester General Hospital Leicester UK; ^3^ St. Vincent's University Hospital University College Dublin Dublin Ireland; ^4^ Department of Psychology University of Southern Denmark Odense Denmark; ^5^ Steno Diabetes Centre Odense (SDCO) Odense Denmark; ^6^ Department of Health and Caring Sciences Western Norway University of Applied Sciences Bergen Norway; ^7^ King's College London London UK

## 1 INTRODUCTION

Continuous glucose monitoring (CGM) systems are now standard of care for people with insulin‐treated diabetes, with time below range (TBR) and frequency of sensor‐detected hypoglycaemia (SDH) accepted metrics quantifying hypoglycaemic exposure and risk, in research outcomes and for clinical purposes.^1^ Whilst much interest is focused on TBR as a cumulative summary of hypoglycaemia exposure, recent work has highlighted the impact of the frequency of hypoglycaemic episodes, with up to 65% being asymptomatic whilst many symptomatic ones are not associated with a low sensor value.^2^

TBR measures time below hypoglycaemia thresholds without any regard to the duration or number of individual episodes. The impact of multiple short episodes may be greater on quality of life than a single prolonged episode; on the other hand, a single prolonged episode might have a more negative impact on cardiovascular risk. The number of episodes can affect the epinephrine response to hypoglycaemia.^3^

Russell and Beck defined SDH as 15 consecutive minutes with a sensor glucose value below a threshold, such as <3.9 mmol/L. The end of an episode was defined as a minimum of 15 consecutive minutes with a sensor glucose ≥3.9 mmol/L and ≥0.55 mmol/L (≥10 mg/dL) above the nadir of the episode (the latter coming into play if the nadir is 3.4–3.8 mmol/L), with at least two sensor values ≥3.9 mmol/L for ≥15 minutes apart with no intervening values <3.9 mmol/L.^4^

Research groups and organisations have not used consistent definitions for SDH as shown in Table 1.^5^, ^6^, ^7^, ^8^, ^9^, ^10^, ^11^ In 2016, the International Hypoglycaemia Study Group (IHSG) defined level 1 (L1) SDH as a glucose alert between ≤3.9 mmol/L and ≥3.0 mmol/L, and level 2 (L2) SDH as <3.0 mmol/L, for a minimum duration per episode (Δt_min_) ≥20 min.^5^ In 2017, the Advanced Technologies and Treatments for Diabetes (ATTD) group used the same thresholds for L1 and L2 SDH, but for a Δt_min_ ≥15 min. The glucose value of 3.9 mmol/L, although included in the IHSG definition of L1 SDH, was excluded by ATTD, to avoid duplicating the value of 3.9 mmol/L as both the lower end of time in range (TIR) and the upper end of L1 hypoglycaemia alert.^6^ The end of a hypoglycaemic episode was defined as a sensor glucose ≥3.9 mmol/L for 15 min.^6^ In its 2019 recommendations for the use of continuous glucose monitoring (CGM), there was no change in the suggested thresholds or minimum duration per episode.^7^ The American Diabetes Association (ADA) referenced the 2019 ATTD consensus guidelines on CGM in its 2024 standards of care of diabetes.^8^

**TABLE 1:** Comparison of different SDH definitions in terms of glucose thresholds and minimum duration below threshold among international consensus guidelines.

	SDH level	Glucose threshold	Δt_min_ below threshold	End of hypoglycaemic episode
UK Hypoglycaemia Study Group 2007^10^	‘Level 1’	N/A	N/A	N/A
	‘Level 2’	<3.0 mmol/L	20 min	Sensor reading for 20 min above the respective threshold
IHSG 2016^5^	Level 1	≤3.9 and ≥3.0 mmol/L	20 min	Absent
	Level 2	<3.0 mmol/L	20 min	
ATTD 2017^6^	Level 1	<3.9 and ≥3.0 mmol/L	15 min	Sensor reading for 15 min at ≥3.9 mmol/L
	Level 2	<3.0 mmol/L	15 min	
HypoDE 2018^11^	Level 1	<3.9 and >3.0 mmol/L	20 min	Absent
	Level 2	<3.0 mmol/L	20 min	Absent
ATTD 2019^7^	Level 1	<3.9 and ≥3.0 mmol/L	20 min	Absent
	Level 2	≤3.0 mmol/L	20 min	
CONCLUDE 2020^9^	Level 1	N/A	N/A	Absent
	Level 2	≤3.0 mmol/L	Absent	
ADA 2024^8^	Level 1	<3.9 and ≥3.0 mmol/L	15 min	Absent
	Level 2	<3.0 mmol/L	15 min	

We explored the impact of different definitions (ADA, ATTD, IHSG) and different thresholds (≤3.9 mmol/L vs. ≤3.8 mmol/L) and Δt_min_ = 15 min versus 20 min on TBR and the median number of weekly L1 and L2 SDH episodes in people with insulin‐treated diabetes.

## 2 METHODS

### 2.1 Study design

We analysed data collected during the Hypo‐METRICS study.^2^ This was a 10‐week observational study of people with type 1 (T1D) or insulin‐treated type 2 diabetes (T2D), during which participants wore a blinded CGM device (Abbott Freestyle Libre 2) throughout, while continuing their usual form of glucose monitoring.

### 2.2 Study population

Participants were aged 18–85 years and had experienced at least one hypoglycaemic episode in the month preceding enrolment. Participants were excluded if eGFR was <30 mL/min/1.73 m^2^ or if they used automated insulin delivery systems.

### 2.3 Statistical analysis

We performed t‐tests to compare the number of SDH episodes using ADA, ATTD, and IHSG definitions, and calculated the impact of different thresholds (≤3.8 mmol/L vs. ≤3.9 mmol/L) and different durations (15 min vs. 20 min) at L1 and L2 on TBR and rates of episodes/week. Bonferroni correction was applied to adjust for multiple comparisons.

## 3 RESULTS

We used data from 599 participants (54% T2D), median (IQR) age = 56 years. (20), HbA1c = 7.37% (1.27%), 57% routine CGM use, 55% male, 89% white.

There was no statistically significant difference in the number of weekly episodes at ≤3.8 mmol/L with a Δt_min_ = 15 min (ATTD/ADA definitions) versus ≤3.9 mmol/L with a Δt_min_ = 20 min (IHSG definition): 5.14 versus 4.76, *p* = 0.91. TBR was also not different at ≤3.8 mmol/L versus ≤3.9 mmol/L (3.86% vs. 4.05%, *p* = 0.99).

**FIGURE 1:**
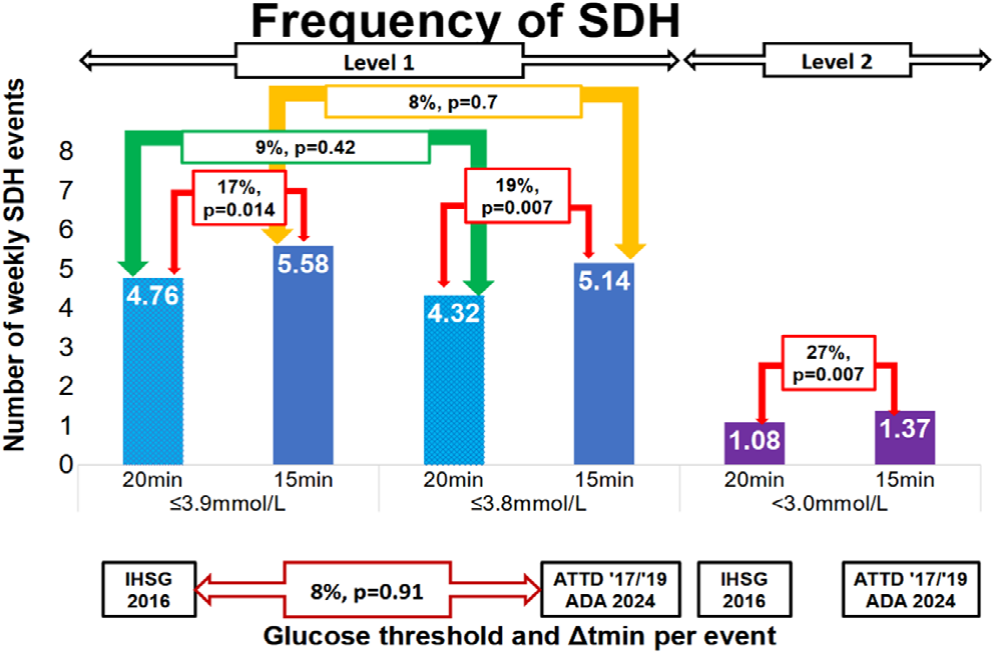
SDH international guidance definitions according to different glucose thresholds and minimum duration.

Duration had a significant impact on the number of episodes (Figure 1). When comparing the shorter (15 min) versus the longer duration (20 min), at ≤3.8 mmol/L there were 19% more episodes (5.14 vs. 4.32, *p* = 0.007); at ≤3.9 mmol/L there were 17% more SDH episodes (5.58 vs. 4.76, *p* = 0.014), and at <3.0 mmol/L there were 27% more episodes (1.37 vs. 1.08, *p* = 0.007). There were no statistically significant differences in the number of SDH episodes when comparing a threshold of ≤3.8 mmol/L to ≤3.9 mmol/L, regardless of the minimum duration.

## 4 CONCLUSIONS

This analysis showed that changing the upper threshold for defining L1 SDH (as happened in 2017) did not significantly affect the median number of SDH episodes/week or TBR; changing the minimum duration did have a significant impact. Defining the minimum duration of hypoglycaemic episodes as ≥15 min under the threshold yielded 19% and 17% more L1 episodes at ≤3.8 mmol/L and ≤3.9 mmol/L respectively, with 27% more L2 episodes at <3.0 mmol/L when compared to a minimum duration per episode set at 20 min under the threshold.

The lack of significant differences in TBR or rates of hypoglycaemia between the IHSG and ATTD definitions might be because the reduced threshold balanced out the reduced duration.

Whilst TBR provides an overall summary of hypoglycaemia exposure, it does not necessarily reflect the risk of severe hypoglycaemia (SH). Canha et al. have demonstrated that high TBR increases SH risk only in the context of impaired awareness.^12^

The ATTD, despite its 2017 recommendations that hypoglycaemia should be quantified as (a) TBR and (b) number of hypoglycaemic episodes over the CGM reporting period, has not included the latter in its 2019 consensus on CGM data interpretation^6^, ^7^; frequency of hypoglycaemia is not currently reported in CGM metrics.

Strengths of this analysis are the high CGM data completion rate (>90%), with approximately 1 million hours of CGM data collected, and with a median active user time of 95.1% (IQR = 9.7%). We expect any intrapersonal sensor inaccuracies due to exercise, compression artefacts or lower accuracy on the first day of sensor usage to even out. These data were collected in a predominantly white European population, using a Freestyle Libre, so the estimations may vary slightly in more diverse populations or with different sensors. Recent data suggesting discrepancies between estimates of TBR with different meters^13^ may have reduced our absolute rates of hypoglycaemia but as the same meter was used throughout, the relationships we describe remain valid.

It is likely that factors such as age, sex, or the type of diabetes affect the frequency and/or duration of SDH events. For example, preserved C‐peptide/glucagon secretion is associated with fewer SDH episodes, which may indicate different physiological counterregulation responses to hypoglycaemia between T1D and T2D; however, as international organisations have not accounted for such variables in their definitions, neither have we.^14^ This analysis does not help identify what the correct thresholds and/or duration of sensor‐detected hypoglycaemia that best correlate with biological and psychosocial adverse effects are, but it does highlight that using slightly different definitions can have impacts on results.

In conclusion, refining the threshold for L1 hypoglycaemia from ≤3.9 mmol/L (IHSG) to <3.9 mmol/L (ATTD/ADA) does not impact TBR. Duration per episode has a larger impact on the frequency of SDH than the threshold: we recommend that the rate of SDH be reported alongside TBR. Further research is required in determining the minimum duration that has an impact on inflammatory, counterregulatory, and symptomatic responses to hypoglycaemia.

## AUTHOR CONTRIBUTIONS

Vaios Koutroukas and Pratik Choudhary developed the plan for the manuscript, with input and advice from the remaining coauthors. Jonah Thomas conducted the statistical analysis and generated the demographics table. Vaios Koutroukas prepared the manuscript draft, with critical feedback from the remaining coauthors. Jonah Thomas, Gilberte Martine‐Edith, Patrick Divilly, Claire L. Meek, Frans Pouwer, Stephanie A. Amiel, and Pratik Choudhary prepared subsequent revisions with feedback from all coauthors. All authors approved the final draft of the manuscript. Vaios Koutroukas is the guarantor of this work and, as such, had full access to all the data in the study and takes responsibility for the integrity of the data and the accuracy of the data analysis.

## FUNDING INFORMATION

This work was supported by the Innovative Medicines Initiative 2 Joint Undertaking (JU) under grant agreement 777460. JU receives support from the European Union's Horizon 2020 Research and Innovation Programme and European Federation of Pharmaceutical Industries and Associations and T1D Exchange, Juvenile Diabetes Research Foundation, International Diabetes Federation, and The Leona M. and Harry B. Helmsley Charitable Trust. The industry partners supporting JU include Abbott Diabetes Care, Eli Lilly, Medtronic, Novo Nordisk, and Sanofi‐Aventis. Abbott Diabetes Care provided the continuous glucose monitors used in the study.

The views expressed are those of the author(s) and not necessarily those of the National Health Service, NIHR, the Department of Health and Social Care or the JU.

## CONFLICT OF INTEREST STATEMENT

Vaios Koutroukas has received funding from KelCon GmbH to attend ATTD 2024. He is also the recipient of the YDEF‐Lilly scholarship to attend EASD 2024, and attended the EASD Robert Turner course 2025, partially funded by Lilly. Jonah Thomas has no disclosures to declare. Gilberte Martine‐Edith has no disclosures to declare. Patrick Divilly has spoken at educational symposia sponsored by Novonordisk, Grünenthal, and Menarini Pharmaceuticals (Ireland) and has served on an advisory board for Dexcom. Claire L. Meek is supported by the European Foundation for the Study of Diabetes—Novo Nordisk Foundation Future Leaders' Award (NNF19SA058974). Her work on type 1 diabetes in pregnancy is supported by Diabetes UK under project grant number 22/0006456. She has received research funding and equipment from Dexcom Inc. Frans Pouwer has received funding for research from Novo Nordisk, Eli Lilly, and Sanofi‐Aventis Deutschland GmbH. Stephanie A. Amiel has served on an advisory board for Vertex Pharmaceuticals and has spoken at educational symposia sponsored by Vertex and by Sanofi. No other potential conflicts of interest relevant to this article were reported. Pratik Choudhary has received personal fees from Abbott Diabetes Care, Insulet, Dexcom, Novo Nordisk, AstraZeneca, Medtronic, Roche Diabetes Care, and Sanofi Diabetes and research funding support from Abbott Diabetes Care, Medtronic, and Novo Nordisk.

## Supporting information


**Data S1.** Supporting Information.

## Data Availability

The data that support the findings of this study are available from the corresponding author upon reasonable request.
