# Supplementary material for: Exploring the impact of different definitions of level 1 and level 2 sensor‐detected hypoglycaemia upon frequency of hypoglycaemia and time below range: The Hypo‐METRICS study
Source: Diabetes Obes Metab. 2025 Oct 27;28(1):775–8. doi: 10.1111/dom.70238 (PMC12673437; doi:10.1111/dom.70238)
Supplement: Supplementary file 1 — Data S1. Supporting Information. [file DOM-28-775-s001.docx]

**Supplementary appendix**. Summary of demographic characteristics of the study population.

| **Variable** | **Study sample (n = 599)** |
| --- | --- |
| **Sex**: male, n (%) | 329 (55) |
| **Age** (yr): median (IQR) | 56 (20) |
| **Type 2 diabetes**, n (%) | 323 (54) |
| **Ethnicity**, n (%) |  |
| White | 533 (89) |
| Other ethnicity/did not disclose | 66 (11) |
| **Diabetes duration** (yr): median (IQR) | 19 (16) |
| **HbA1c** (%), median (IQR) | 7.37 (1.27) |
| **Glucose monitoring**, n (%) |  |
| CGM | 341 (57) |
| CBG | 258 (43) |
| **CGM time sensor active**: median (%), (IQR) | 95.1 (9.7) |
| **Awareness status**, n (%)* |  |
| Intact awareness | 454 (76) |
| Impaired awareness* | 145 (24) |

*Impaired awareness defined as Gold score ≥4.
